# ADAMTS and ADAM metalloproteinases in osteoarthritis – looking beyond the ‘usual suspects’

**DOI:** 10.1016/j.joca.2017.02.791

**Published:** 2017-07

**Authors:** C.-Y. Yang, A. Chanalaris, L. Troeberg

**Affiliations:** Arthritis Research UK Centre for Osteoarthritis Pathogenesis, Kennedy Institute of Rheumatology, Nuffield Department of Orthopaedics, Rheumatology and Musculoskeletal Sciences, University of Oxford, Roosevelt Drive, OX3 7FY Oxford, UK

**Keywords:** Osteoarthritis, Metalloproteinase, ADAMTS, ADAM

## Abstract

**Introduction:**

Matrix metalloproteinases (MMPs) and ‘aggrecanase’ a disintegrin and metalloproteinase with thrombospondin motifs (ADAMTSs) are well established to play key roles in osteoarthritis (OA) through degradation of extracellular matrix (ECM) type II collagen and aggrecan, and are thus potential targets for development of OA therapies.

**Objective:**

This paper aims to provide a comprehensive review of the expression and potential roles of other, lesser-known ADAMTSs and related adamalysins (or a disintegrin and metalloproteinases (ADAMs)) in cartilage, with a view to identifying potentially protective or homeostatic metalloproteinases in the joint and informing consequent selective inhibitor design.

**Design:**

A comprehensive literature search was performed using PubMed terms ‘osteoarthritis’ and ‘ADAMTS’ or ‘ADAM’.

**Results:**

Several ADAMTSs and ADAMs were identified as having reportedly increased expression in OA. These include enzymes likely to play roles in cartilage matrix anabolism (e.g., the procollagen N-proteinases ADAMTS-2, ADAMTS-3 and ADAMTS-14), chondrocyte differentiation and proliferation (e.g., ADAM9, ADAM10, ADAM12), as well as enzymes contributing to cartilage catabolism (e.g., Cartilage oligomeric protein (COMP)-degrading ADAMTS-7 and ADAMTS-12).

**Conclusions:**

In addition to the well-characterised MMPs, ADAMTS-4 and ADAMTS-5, many other ADAMTSs and ADAMs are expressed in cartilage and several show significantly altered expression in OA. Studies aimed at elucidating the pathophysiological roles of these enzymes in cartilage will contribute to our understanding of OA pathogenesis and enable design of targeted inhibitors that effectively target metalloproteinase-mediated cartilage degradation while sparing cartilage repair pathways.

## Introduction

Osteoarthritis (OA) is a common degenerative joint disease characterised by cartilage loss, subchondral bone remodelling and osteophyte development. These structural changes are accompanied by impaired movement, stiffness and chronic joint pain. Primary risk factors for OA include age, obesity and joint injury, which alter the mechanical loading of the joint and initiate dysregulated cellular signalling and activation of catabolic pathways.

The role of matrix metalloproteinases (MMPs) in osteoarthritic degradation of the extracellular matrix (ECM) has been well documented. In particular, the collagenase matrix metalloproteinase 13 (MMP-13) plays a central role in degrading type II collagen^1^, ^2^, and the two ‘aggrecanases’, namely a disintegrin and metalloproteinase with thrombospondin motifs (ADAMTS)-4 and -5, degrade aggrecan^3^, ^4^. Collagen and aggrecan are the primary structural components of the cartilage ECM, and their degradation correlates with progression of OA. Collagenases and aggrecanases are thus potential targets for the development of disease-modifying OA drugs (DMOADs). For such an approach to be successful, it is vital that we learn lessons from previous attempts to develop metalloproteinase inhibitors as anti-cancer therapies^5^. These drugs failed due to limited specificity and consequent off-target inhibition of other metalloproteinases with homologous catalytic domains. Only by understanding the full spectrum of metalloproteinases expressed in the joint and their biological function(s) in this location will it be possible to design strategies to selectively target pathological tissue destruction. Several ADAMTSs other than ADAMTS-4 and -5 are expressed in cartilage, but little is known about whether they are required for joint health or whether they contribute to OA pathogenesis. The roles of the related adamalysin (a disintegrin and metalloproteinase, ADAM) family of metalloproteinases in cartilage are similarly poorly understood. Here, we review studies examining the role of ADAMTSs and ADAMs in cartilage, and compare microarray studies examining their expression in murine models of OA^6^, ^7^, ^8^ and human normal and osteoarthritic cartilage^9^, ^10^, ^11^, ^12^, ^13^, ^14^, ^15^, ^16^, ^17^. This review thus highlights ADAMTSs and ADAMs that are expressed in cartilage and whose expression is altered in OA, with a view to developing a broader understanding of the contribution of the metalloproteinase family to joint health and disease.

## ADAMTSs

**Fig. 1:**
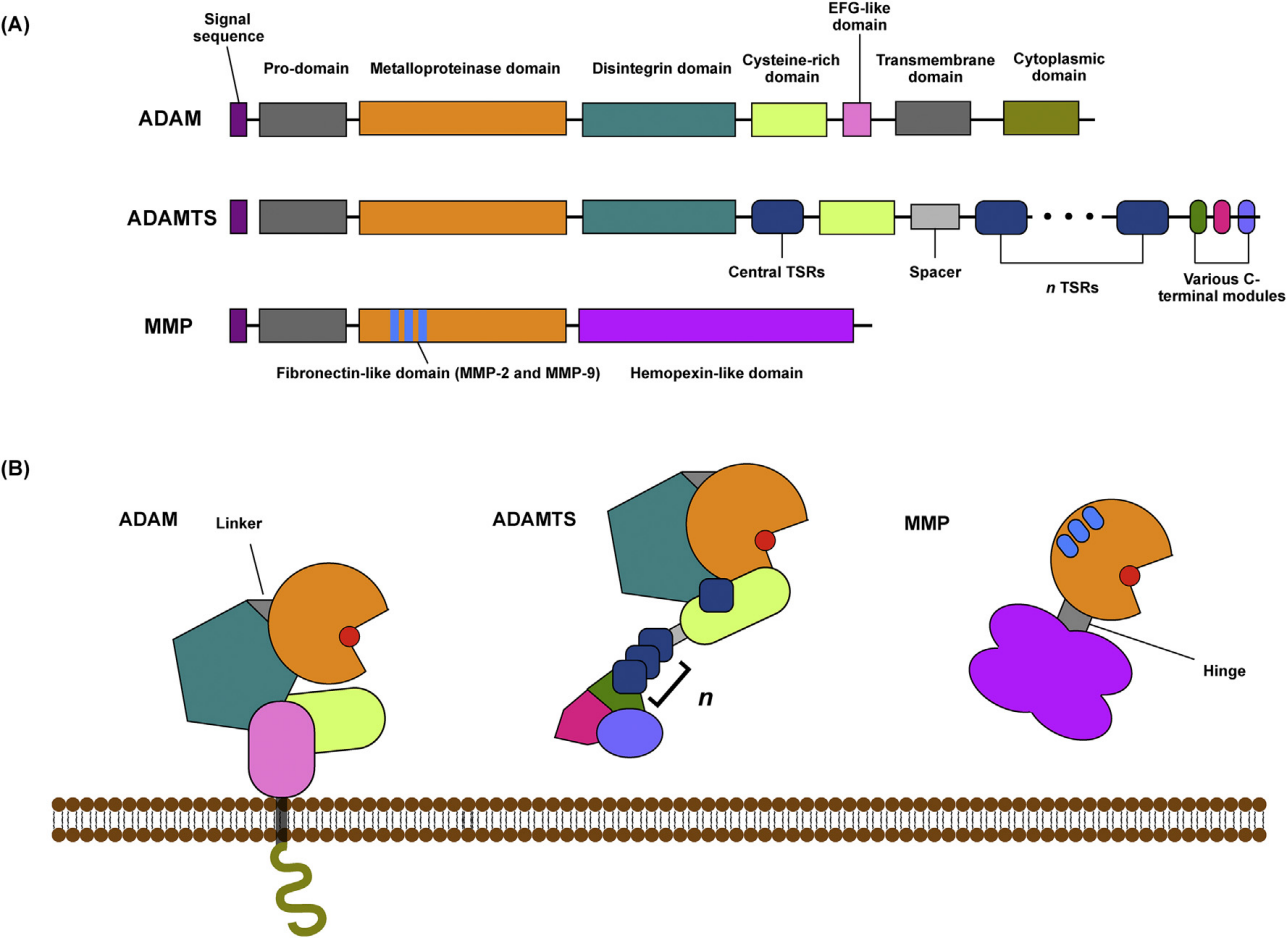
Schematic representation of ADAM and ADAMTS topography. ADAMs and ADAMTSs are metzincin metalloproteinases whose catalytic domains share homology with those of the MMPs, and contain a zinc ion (red circle) that is essential for their proteolytic activity. All three groups of enzymes have a prodomain that keeps them in an inactive zymogen form until they are activated. The families differ in their C-terminal ancillary domains, which mediate interaction with substrates and other proteins. *ADAM ancillary domains*: ADAMs contain C-terminal disintegrin-like domains, thought to regulate cell–cell and cell–matrix adhesion, as well as conserved cysteine-rich domains and EGF-like domains^102^. The cytoplasmic domains are the most diverse, and vary in sequence and length. Some ADAM cytoplasmic domains contain proline-rich Src homology (SH)-2 and/or SH-3 binding sites, indicating that they may participate in intracellular signalling. Some also contain potential serine–threonine and/or tyrosine phosphorylation sites, making them plausible adaptors for conveying signals between the cell and its surroundings. *ADAMTS ancillary domains*: In contrast to the ADAMs, ADAMTSs are secreted metalloproteinases that lack transmembrane and cytoplasmic domains. In addition to their catalytic and pro-domains, the enzymes contain a variable number of thrombospondin type 1 sequence repeat (TSR) motifs, which are homologous to thrombospondins^18^, as well as a cysteine-rich domain and spacer domain. Some members of the family contain additional C-terminal domains^18^. For example, ADAMTS-9 and -20 contain GON-1 domains, ADAMTS-2, -3 and -14 contain a procollagen N-propeptidase (PNP) domain, and ADAMTS-7 and -12 contain a PLAC domain.

The ADAMTSs are a family of 19 secreted metalloproteinases (Fig. 1) involved in various developmental and homeostatic processes^18^. The ‘aggrecanases’ ADAMTS-4 and -5 have been extensively reviewed elsewhere^19^, ^20^, and will not be covered here. Several other ADAMTSs are expressed in cartilage, and have emerging roles in joint pathophysiology.

### ADAMTS-1

**Fig. 2:**
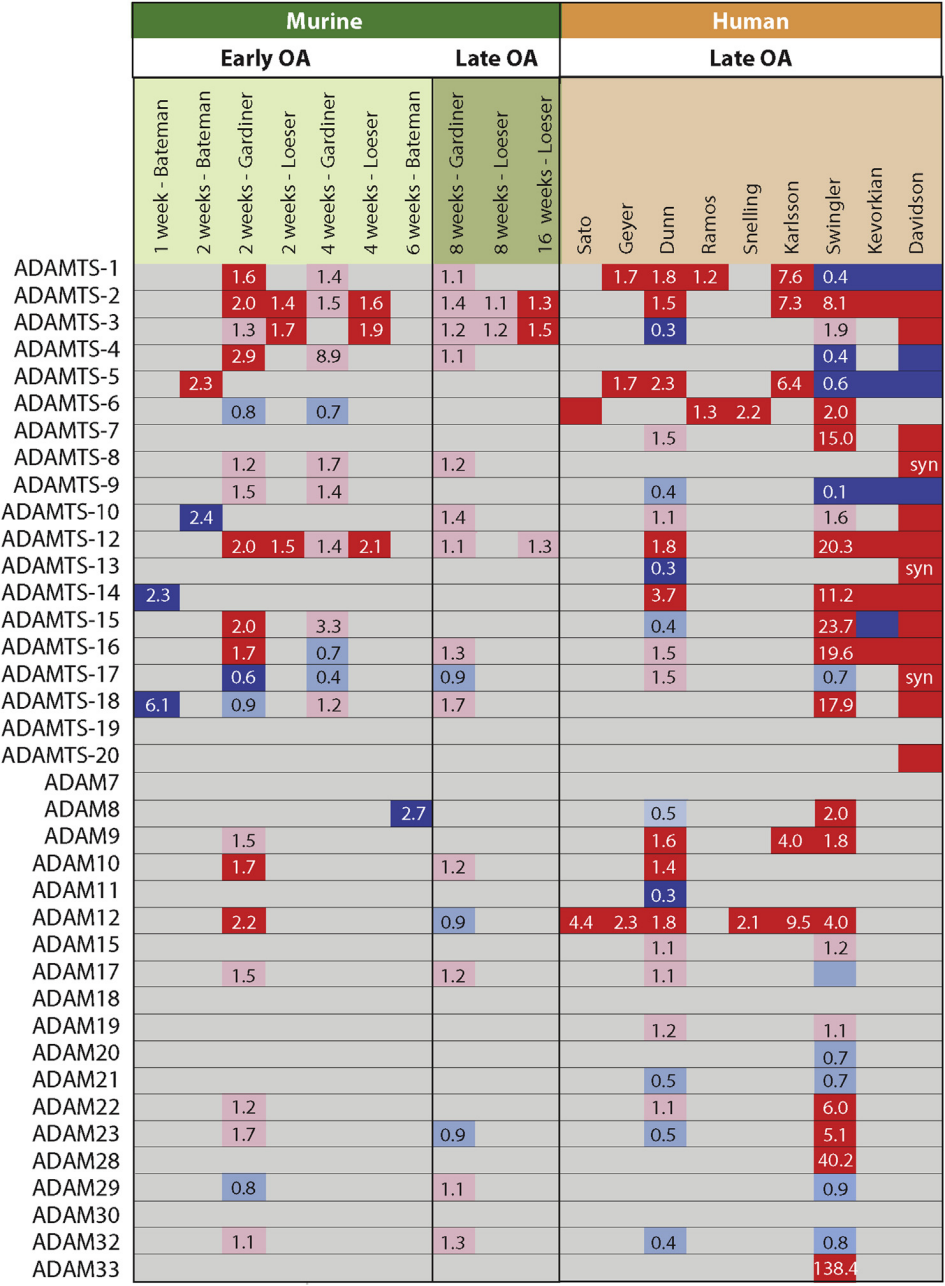
Fold-change in expression of ADAMTSs and ADAMs in OA compared to normal cartilage. Upregulated genes are marked in red (statistically significant, *P* < 0.05) or pink (not statistically significant, *P* > 0.05), while down-regulated genes are shown in dark blue (statistically significant, *P* < 0.05) or light blue (not statistically significant, *P* > 0.05). Bateman^6^, Gardiner^7^ and Loeser^8^ analysed murine knee cartilage at various time points after DMM. Sato^15^, Geyer^11^, Dunn^10^, Ramos^14^, and Snelling^16^ analysed paired samples from intact and OA lesion areas of the same patients. Karlsson^12^ compared knee OA samples with healthy controls. Swinger^17^, Kevorkian^13^ and Davidson^9^ compared femoral head cartilage from OA patients with that of fracture patients. Syn, synovium.

ADAMTS-1 is expressed in cartilage and synovium^18^ and has been shown to cleave aggrecan and versican^21^. Several studies show that ADAMTS-1 expression is significantly upregulated in OA cartilage^7^, ^10^, ^11^, ^12^, ^14^, ^22^, though some studies indicate reduced expression in late-stage human OA^9^, ^13^, ^17^ (Fig. 2). Immunohistochemical analysis indicates that in normal cartilage, ADAMTS-1 is primarily expressed in the superficial zone, with OA cartilage showing increased staining in the middle zone and in osteophytes^22^.

*Adamts1*-null mice display unaltered susceptibility in the antigen-induced model of inflammatory arthritis and there is also no change in the level of aggrecan degradation in response to interleukin 1 (IL-1) stimulation of cartilage explants *in vitro*^23^. The susceptibility of these mice to surgical destabilisation of the medial meniscus (DMM), a model that more closely resembles human OA, has not been reported. Given that *Adamts1*-null mice show developmental abnormalities^24^, conditional deletion may be required to establish the role of the enzyme in cartilage homeostasis.

### ADAMTS-2, ADAMTS-3 and ADAMTS-14

ADAMTS-2, ADAMTS-3 and ADAMTS-14 are procollagen N-proteinases, responsible for removing the N-terminal propeptide of type I, II, III and V pro-collagen^25^, ^26^, ^27^, and thereby enabling collagen fibril formation. Lack of procollagen N-proteinase activity is associated with defective collagen fibrilogenesis and connective tissue dysfunctions such as skin fragility^26^. Since type II collagen is a critical structural component of the cartilage ECM, these enzymes are likely to be important for cartilage homeostasis and repair. Bekhouche *et al.*^28^ recently identified additional substrates for this group of enzymes, including several proteins involved in transforming growth factor β (TGFβ) signalling.

Several studies indicate statistically significant increased expression of ADAMTS-2 in OA cartilage^7^, ^8^, ^9^, ^10^, ^12^, ^13^, ^17^. No cartilage or joint abnormalities have been identified in *Adamts2*-deficient mice^26^, although they exhibit skin fragility reminiscent of Ehlers-Danlos syndrome in humans, which is also caused by mutation of ADAMTS-2. Partial processing of collagen in these mice appears to indicate some redundancy between the procollagen N-proteinases.

ADAMTS-3 is more potent than ADAMTS-2 at processing type II pro-collagen^29^, suggesting that it may be more relevant than ADAMTS-2 in cartilage. As with ADAMTS-2, ADAMTS-3 expression is reportedly increased in OA cartilage^8^, ^9^, although this reaches statistical significance in fewer studies than ADAMTS-2, and Dunn *et al.*^10^ report a reduction in expression in late stage human OA cartilage. *Adamts3* knockout mice are not viable after E15.0^30^, so generation of conditional knockout mice will be necessary to investigate the role of this enzyme in adult cartilage homeostasis and OA pathogenesis.

ADAMTS-14, the third of the procollagen N-proteinases to be discovered^31^, is also significantly up-regulated in human OA cartilage^9^, ^10^, ^13^, ^17^. Single nucleotide polymorphisms (SNPs) of *ADAMTS14* have been associated with an increased risk of knee OA in two female cohorts^32^, ^33^. The enzyme may be differently regulated in murine OA models, since little regulation is observed in most murine microarray studies, other than reduced expression observed by Bateman *et al.*^6^ 1 week after surgical induction of OA in mice.

The increased expression of all three of these enzymes in human OA cartilage may reflect an attempted anabolic repair response of osteoarthritic chondrocytes, and it would be desirable to spare these enzymes when designing metalloproteinase inhibitors for potential OA therapy.

### ADAMTS-7 and ADAMTS-12

ADAMTS-7^34^ and ADAMTS-12^35^ are thought to contribute to OA pathogenesis by degrading cartilage oligomeric protein (COMP), an important regulator of cartilage ECM assembly and a potential biomarker for cartilage degradation.

Several studies have observed increased expression of ADAMTS-7 in OA cartilage^9^, ^17^, ^23^, ^36^, ^37^, ^38^. OA progression and COMP degradation were increased in mice over-expressing ADAMTS-7 and decreased in *Adamts7*^−/−^ mice^37^. COMP degradation could also be inhibited *in vitro* by addition of ADAMTS-7 neutralising antibodies or siRNA^39^. Expression of the enzyme can be stimulated by pro-inflammatory cytokines such as tumour necrosis factor (TNF)^39^. A positive feedback loop has been proposed between ADAMTS-7 and TNF, since TNF expression is elevated in transgenic mice over-expressing ADAMTS-7 in chondrocytes^37^. The molecular mechanism underpinning this feedback loop is not known.

Increased expression of ADAMTS-12 is also consistently observed in OA cartilage^7^, ^8^, ^9^, ^10^, ^13^, ^17^, ^35^, ^38^. COMP degradation in OA cartilage explants could be additively inhibited by neutralising antibodies against ADAMTS-7 and ADAMTS-12^39^, suggesting that ADAMTS-12 may also contribute to pathological COMP degradation. These enzymes may also work together to process substrates *in vivo*. Systematic analysis of single and combined *Adamts7-*and *Adamts12*-null mice would help to clarify the relative contributions these enzymes make to COMP degradation in OA.

### ADAMTS-8

ADAMTS-8 is expressed in normal cartilage and has been reported to have aggrecanase activity^40^. Expression of the enzyme in cartilage is not significantly altered in murine models of OA,^7^, ^15^ although Davidson *et al.*^9^ reported increased expression in human OA synovium.

### ADAMTS-9 and ADAMTS-20

ADAMTS-9 and ADAMTS-20 are the largest members of the ADAMTS family, with 15 thrombospondin domains and a C-terminal GON-1 domain.

ADAMTS-9 is expressed in normal cartilage, and highly induced in response to pro-inflammatory cytokines (e.g., IL-1β and TNF^41^) and adipokines (e.g., leptin^42^). Expression is reduced in late-stage OA cartilage^9^, ^13^, ^17^. ADAMTS-9 is able to cleave aggrecan, although a truncated form of the enzyme (comprising the catalytic, disintegrin and first TS domain) exhibits low aggrecanase activity compared to a similarly truncated form of ADAMTS-5^43^. It is not known whether the full-length enzyme has higher aggrecanase activity, since this large enzyme is difficult to express and purify.

ADAMTS-20 is expressed in cartilage and reportedly shows increased expression in OA^9^, but the enzyme is primarily considered to be important for versican cleavage during development.

### ADAMTS-15

ADAMTS-15 is also thought to participate in developmental versican cleavage^44^, but is expressed in cartilage and can cleave aggrecan^45^. Three microarray studies found increased expression of ADAMTS-15 in OA cartilage^7^, ^9^, ^17^, while two^10^, ^13^ found reduced expression. The contribution of the enzyme to cartilage homeostasis and disease is unknown.

### ADAMTS-16

ADAMTS-16 expression is increased in OA cartilage^7^, ^9^, ^13^, ^17^ and a truncated form of the enzyme shows some aggrecanase activity^43^. To our knowledge, the aggrecanase activity of the full-length enzyme has not been characterised. Surridge *et al.*^46^ observed that over-expression of ADAMTS-16 in SW1353 chondrosarcoma cells decreased *MMP13* expression, cell migration and proliferation, raising the possibility that ADAMTS-16 may have a protective role. Mechanistic investigation of these observations may shed light on the role of this enzyme in cartilage.

### Other ADAMTSs

Microarray studies have reported increased expression of ADAMTS-6^14^, ^15^, ^16^, ^17^, ADAMTS-10^9^, and ADAMTS-18^9^, ^17^ in OA cartilage. These are ‘orphan’ enzymes without known substrates. Studies on their wider biological functions have implicated ADAMTS-6 and -10 in regulations of cell–cell junctions^47^ and ADAMTS-18 in development^48^.

### ADAM metalloproteinases

The **A D**isintegrin **a**nd **M**etalloproteinase (ADAM, or adamalysin) family are conserved type-I transmembrane metzincin metalloproteinases related to the MMPs and ADAMTSs (Fig. 1). ADAMs are widely expressed and have been shown to participate in a wide variety of biological processes^49^, ^50^.

Among the 34 known ADAMs, 20 are present in the human genome and 12 of these (ADAM8, ADAM9, ADAM10, ADAM12, ADAM15, ADAM17, ADAM19, ADAM20, ADAM21, ADAM28, ADAM30 and ADAM33) are predicted to be proteolytically active based on the presence of a conserved HEXGHXXGXXHD motif and downstream ‘Met turn’ in the catalytic domain^49^. The proteolytically active ADAMs mainly function as ‘sheddases’, cleaving the juxta-membrane region of their trans-membrane substrates (reviewed by Edwards *et al.*^49^) to release the soluble ectodomain of the substrate to the extracellular space. This activity enables them to regulate the extracellular availability of autocrine and paracrine signalling molecules, such as transmembrane cytokines and growth factors and their receptors. The remaining eight human ADAMs (ADAM2, ADAM7, ADAM11, ADAM18, ADAM22, ADAM23, ADAM29, ADAM32) are predicted to lack proteolytic activity, but still play important biological roles^49^.

The roles of ADAMs in development of the musculoskeletal system have been well documented, and the roles of some of the ADAMs in rheumatoid arthritis have been examined. The roles of these enzymes in adult joint tissues and in OA have been less well characterised. We review the emerging evidence for roles of these enzymes in joint homeostasis and OA pathogenesis.

### ADAM8

Expression of ADAM8 is reportedly elevated in human OA cartilage^17^, ^51^, ^52^, and the enzyme has been postulated to contribute to OA pathogenesis by cleaving fibronectin and generating cryptic pro-catabolic fibronectin fragments^52^. Zack *et al.*^52^ showed that addition of recombinant ADAM8 to human OA cartilage explants increases aggrecan cleavage and cartilage degradation *in vitro*. Expression of ADAM8 was significantly reduced 6 weeks after surgical induction of OA in a murine model^6^, indicating either species-specific differences in regulation or that the enzyme is dynamically regulated during OA progression.

ADAM8 is also expressed in cells of the mononuclear phagocyte lineage^53^. ADAM8 promotes osteoclast formation^54^, ^55^ and is thought to contribute to the bone erosion associated with aseptic loosening of hip replacement prostheses^56^. Increased expression of ADAM8 has been observed in rheumatoid arthritis pannus tissue^55^, suggesting that ADAM8 may also contribute to osteoclast formation and bone erosion in rheumatoid arthritis.

### ADAM9 (meltrin-Ɣ)

Several studies consistently report significant upregulation of ADAM9 in human OA cartilage^10^, ^12^, ^17^ and the enzyme is non-significantly upregulated in mice 2 weeks after DMM surgery^7^. ADAM9 substrates include growth factor precursors^49^, and the enzyme has been suggested to play a role in chondrogenesis^57^. Its expression is decreased in response to IL-1 or retinoic acid^58^. Reminiscent of ADAM8, ADAM9 is expressed in mononuclear phagocytes and osteoclasts, and has been suggested to contribute to bone resorption associated with aseptic loosening of hip prostheses^59^.

### ADAM10

ADAM10 is one of the best characterised of the ADAMs, and is crucial for embryonic development through its shedding of Notch receptor and consequent regulation of downstream Notch signalling^49^. ADAM10 also has pivotal roles in cell migration and adhesion, mediated through its cleavage of transmembrane precursors of growth factors (e.g., epidermal growth factor, EGF), chemokines (e.g., CX3CL1 and CXCL16) and adhesion molecules (e.g., E-cadherin and VE-cadherin)^49^.

Expression of ADAM10 is low in normal adult cartilage, but increased during development^60^, in OA^7^, ^10^, ^60^ and in response to pro-inflammatory cytokines^60^, ^61^. In OA or IL-1α-stimulated cartilage, highest expression of ADAM10 was observed in regions with greatest damage and proteoglycan loss^60^, leading Chubinskaya *et al.*^60^ to suggest the enzyme may contribute to cartilage damage. ADAM10 substrates in normal or OA cartilage have not been characterised.

ADAM10 expression is also increased in synovium lining and endothelial cells of rheumatoid arthritis patients^61^. Silencing of ADAM10 in endothelial cells reduced migration and tubule formation, suggesting the enzyme may contribute to angiogenesis in the rheumatoid synovium^61^.

### ADAM12 (meltrin-α)

ADAM12 is the ADAM most consistently reported to display increased expression in human OA cartilage^10^, ^11^, ^12^, ^15^, ^16^, ^17^, ^62^. Its expression is reported to correlate with Mankin score^62^, and increased expression has also been reported in mice 2 weeks after DMM surgery^7^. Several studies have linked ADAM12 SNPs (e.g., rs1278279, rs3740199, rs1044122, and rs1871054) with an increased risk of OA^63^, ^64^, ^65^, ^66^, ^67^, ^68^, ^69^, ^70^, although post-hoc stratification of data was often required to establish a significant association. A large case-controlled study of over 1000 UK OA patients and an equal number of matched controls failed to find any association between ADAM12 SNPs and OA^71^.

Substrates of ADAM12 in cartilage have not been directly investigated. The enzyme is widely expressed and has been shown to promote cell proliferation, differentiation and migration through its ability to shed transmembrane ligands of the EGF receptor and thus stimulate EGF receptor signalling^72^. ADAM12 also modulates insulin-like growth factor (IGF) signalling by cleavage of IGF binding proteins^72^. ADAM12 has similarly been shown to promote chondrocyte proliferation and maturation during development^62^, ^73^, although it is proposed to act by promoting IGF-1 activity by degrading IGF binding protein 5^62^, rather than by an EGF receptor-dependent pathway.

### ADAM15 (Metargidin)

ADAM15 is the only ADAM so far demonstrated to have a protective role in cartilage, with *Adam15*-deficient mice developing accelerated spontaneous OA with age^74^. Bohm *et al.* reported that expression of the enzyme is increased in OA cartilage using *in situ* hybridisation^75^, but subsequent microarrays have reported either no change^7^ or slight but statistically non-significant increased expression in OA^10^, ^17^.

Understanding of how ADAM15 protects cartilage is still in its infancy. Böhm *et al.* proposed that ADAM15 increases chondrocyte survival by reinforcing adhesion to collagen types II and VI^74^, promoting outside-in pro-survival signalling^76^, ^77^ and up-regulating anti-apoptotic molecules such as X-linked inhibitor of apoptosis (XIAP)^78^.

ADAM15 has been shown to affect cell–cell and cell–matrix adhesion in other cell types^79^, ^80^, ^81^, potentially also through its ability to interact with integrins. ADAM15 is the only ADAM that contains an integrin-binding Arginine-glycine-aspartic acid (RGD) motif in its disintegrin domain^82^, enabling RGD-dependent interaction with α_v_β_3_ and α_5_β_1_^83^, and ADAM15 is also able to bind to α_9_β_3_ in an RGD-independent manner^84^. ADAM15 over-expression been shown to promote migration of mesangial cells^85^, possibly by disrupting integrin-ECM interactions or by proteolytic cleavage of adhesion molecules. N- and E-cadherin^86^, ^87^ are among the few ADAM15 substrates described, and their degradation may underpin the effect of ADAM15 on cell migration. The relevance of these studies to ADAM15's protective role in cartilage is unclear, especially since many were done using overexpression systems that may not accurately reflect the physiological activity of the enzyme.

It is not yet known how these different activities of ADAM15 are regulated or coordinated. Additionally, ADAM15 substrates in chondrocyte have not been identified. Further studies to investigate the protective role of this enzyme in the joint are required.

### ADAM17 (TNF-α-converting enzyme, TACE)

ADAM17, or TACE, is the most extensively studied ADAM, with important roles in development and inflammation through its shedding of EGF receptor ligands and the membrane-bound precursor of the pleiotropic cytokine TNF^88^.

ADAM17 deletion leads to perinatal lethality, so conditional and tissue-specific deletion have been studied to evaluate the function of the enzyme in specific tissues and in the adult. Deletion of ADAM17 in chondrocytes retarded expansion of hypertrophic chondrocytes in the growth plate and impaired bone growth^89^, ^90^. This indicates that ADAM17 is important for musculoskeletal development, most likely through its role in EGF receptor signalling.

ADAM17 is also expressed in adult chondrocytes^17^, but chondrocyte-specific inducible knockout of ADAM17 has not been reported, so the role of the enzyme in adult cartilage is not known. Microarray studies indicate that ADAM17 expression is not significantly altered in OA^7^, ^10^, ^17^, but such studies do not take account of the fact that ADAM17 activity is largely regulated post-translationally.

Oldefest *et al.*^91^ showed that ADAM17 shedding of the IL-6 receptor (IL-6R) can be inhibited by secreted Frizzled-related protein 3 (sFRP3), but not by variants of sFRP3 that have previously been associated with an increased risk of OA. This raises the possibility that the sFRP3 variants promote cartilage damage by failing to control ADAM17 pro-inflammatory activity in cartilage.

### ADAM19

ADAM19 is expressed during chondrogenesis, with increased expression during the later stages of the process^57^. Expression of the enzyme is reportedly not significantly altered in OA cartilage^10^, ^17^.

### ADAM23

Like ADAM19, ADAM23 is also up-regulated at the late stage of chondrogenesis^57^, ^92^. ADAM23 is up-regulated in OA cartilage^17^, but its function in cartilage has not been investigated.

### ADAM28

ADAM28 is expressed at low levels in normal cartilage, with increased expression in OA^17^. The enzyme has been suggested to promote retinoic acid-stimulated proteoglycan degradation^93^, although the molecular mechanism for this observation has not been established. Known substrates of ADAM28 include TNF^94^ and growth factors such as IGF binding protein-3^95^ and connective tissue growth factor (CTGF)^96^. In osteoblasts, IL-1β-induced MMP-13 expression is dependent on ADAM28^97^, suggesting the enzyme may also affect bone remodelling.

### Other ADAMs

Swinger *et al.*^17^ report that expression of the ADAM22 and ADAM33 is increased in OA cartilage, while expression of ADAM2, ADAM7, ADAM11, ADAM18, ADAM20, ADAM21, ADAM29, ADAM30 and ADAM32 is not significantly altered in OA^17^.

## Concluding remarks and future study directions

Collagenases and aggrecanases have received attention as potential targets for development of DMOADs. In order for such an approach to be successful, we should learn the lessons of previous attempts to design MMP inhibitors to treat cancer, namely that it is crucial to understand the full spectrum of metalloproteinases expressed in a target tissue. Many ADAMTSs and ADAMS are expressed in cartilage and several are differentially regulated in OA, but their roles in cartilage health and disease are largely unexplored and the full spectrum of their substrates is not yet known. Further research into the functions and substrates of these enzymes in the joint and in OA pathogenesis is required to evaluate therapeutic potential. Unbiased proteomic analysis will assist in defining the range of substrates cleaved by each enzyme. Additionally, enzymes may be important at different temporal stages in the disease process, with implications for timing of effective inhibitor therapy. Inducible knockout strategies may be useful in defining relevant windows of activity.

It is especially important to understand which enzymes may serve protective functions in the joint, since inhibiting their activity is likely to further impair cartilage homeostasis. For example, the procollagen N-proteinases ADAMTS-2, ADAMTS-3 and ADAMTS-14 are likely to promote matrix anabolism, and several studies have demonstrated that their expression is increased in OA. Inducible and combined knockout of these enzymes are required to investigate their contribution to cartilage repair in OA and to investigate potential redundancy among the enzymes. Similarly, ADAMs play important roles in signalling in other tissues, and are likely to modulate a diverse range of signalling pathways in chondrocytes. For example, ADAMs can activate signalling pathways by mobilising transmembrane cytokine and growth factor precursors, or through interaction of their cytoplasmic domains with intracellular signalling machinery. Conversely, ADAM-mediated shedding of cytokine and growth factor receptors can suppress their downstream signalling. ADAMs thus fine-tune cellular responses and contribute to maintenance of tissue homeostasis, and changes in ADAM expression may contribute to the shift in balance from cartilage repair to cartilage degradation. Studies on ADAM15, one of the few ADAMs to be studied in the context of OA, have shown that *Adam15-*null animals develop accelerated OA^74^, although the molecular mechanism of this protection is poorly understood. Increased expression of ADAM9, ADAM10 and ADAM12 in OA cartilage has been consistently reported in multiple studies. The activities of these enzymes in other tissues indicate that they may promote chondrocyte differentiation and proliferation, and so may contribute to repair pathways in OA, but direct evidence for this is lacking. Since several of the ADAMs are critically involved in embryogenesis and development, inducible knockout systems will be necessary to expand our understanding of their pathophysiological roles in the joint.

Mechanisms regulating ADAM and ADAMTS expression and activity in the joint also require further study. Several *ADAM* and *ADAMTS* genes displayed altered methylation in OA. These included *ADAMTS2*, *ADAMTS8* (hypermethylated in OA) and *ADAMTS4*, *ADAMTS5*, *ADAMTS10* and *ADAMTS17* (hypomethylated in OA)^98^. How these differences in methylation influence expression is unclear. Expression is also affected by microRNAs, as has been reviewed elsewhere^99^. Additionally, enzymatic activity is often regulated post-translationally. ADAMTS-4 and -5 are post-translationally regulated by endocytosis^100^, and other ADAMTSs may be similarly regulated. Activity of several ADAMs is also regulated post-translationally, through mechanisms such as conformational change, or regulation of substrate or enzyme localisation^101^.

It is important to keep in mind that altered expression of ADAMTSs and ADAMs in OA does not necessarily mean that a particular enzyme is mechanistically involved in disease pathogenesis. This is particularly true in late-stage OA, where expression may be modified by changes in cell homeostasis or altered substrate availability occurring due to matrix catabolism. Knock-out mice studies are required to validate roles in OA pathogenesis. Inducible knock-out models will enable analysis of enzyme functions at different stages of OA progression. Murine studies are potentially complicated by species differences and the question of whether acute surgical models accurately reflect the human disease, but they have the advantage of enabling analysis of early OA, which is difficult to achieve with human clinical samples.

ADAMTSs and ADAMs have the potential to modulate multiple aspects of cell behaviour and tissue homeostasis. Understanding their roles in cartilage is therefore essential for the development of successful therapies to target osteoarthritic cartilage degradation.

## Contributions

CYY and LT performed the literature review and wrote the manuscript. AC analysed microarray data sets.

## Conflict of interest statement

The authors have no financial or personal conflicts of interest.

## Funding

This work was supported by Arthritis Research UK (grants 19466, 20205 and 20887) and the Kennedy Trust for Rheumatology Research.

## Abbreviations

ADAM: a disintegrin and metalloproteinase
ADAMTS: a disintegrin and metalloproteinase with thrombospondin motifs
COMP: cartilage oligomeric protein
CTGF: connective tissue growth factor
DMM: destabilisation of the medial meniscus
DMOAD: disease-modifying OA drug
ECM: extracellular matrix
EGF: epidermal growth factor
IGF: insulin-like growth factor
IL-1: interleukin 1
IL-16R: interleukin 6 receptor
MMP: matrix metalloproteinase
OA: osteoarthritis
PNP: procollagen N-propeptidase
SH: Src homology
SNP: single nucleotide polymorphism
sFRP3: secreted Frizzled-related protein 3
Syn: synovium
TACE: TNF-α-converting enzyme
TGFβ: transforming growth factor β
TNF: tumour necrosis factor
TSR: thrombospondin type 1 sequence repeat
XIAP: X-linked inhibitor of apoptosis protein
